# Predictive performance of CT for adverse outcomes among COVID-19 suspected patients: A two-center retrospective study

**DOI:** 10.17305/bjbms.2020.5466

**Published:** 2021-12

**Authors:** Begumhan Baysal, Mahmut Bilal Dogan, Mutlu Gulbay, Mine Sorkun, Murathan Koksal, Aliye Bastug, Sumeyye Kazancıoglu, Bahadir Orkun Ozbay, Sacit Icten, Ferhat Arslan, Yasemin Cag, Hurrem Bodur, Haluk Vahaboglu

**Affiliations:** 1 Department of Radiology, Istanbul Medeniyet University Goztepe Education and Research Hospital, Istanbul, Turkey; 2 Department of Radiology, University of Health Sciences Ankara City Hospital, Ankara, Turkey; 3 Department of Infectious Diseases and Clinical Microbiology, University of Health Sciences Ankara City Hospital, Ankara, Turkey; 4 Department of Chest Disease, Istanbul Medenıyet Unıversıty Goztepe Education And Research Hospital, Istanbul, Turkey; 5 Department of Infectious Diseases and Clinical Microbiology, Istanbul Medeniyet University Goztepe Education and Research Hospital, Istanbul, Turkey

**Keywords:** COVID-19, pandemic, SARS virus, computed tomography, x-rays

## Abstract

The aim of the study was to compare the performance of various computed tomography (CT) reporting tools, including zonal CT visual score (ZCVS), the number of involved lobes, and Radiological Society of North America (RSNA) categorization in predicting adverse outcomes among patients hospitalized due to the lower respiratory symptoms during the coronavirus disease 2019 (COVID-19) pandemic. A total of 405 patients admitted with severe respiratory symptoms who underwent a chest CT were enrolled. The primary adverse outcome was intensive care unit (ICU) admission of patients. Predictive performances of reporting tools were compared using the area under the receiver operating characteristic curves (AUC ROC). Among the 405 patients, 39 (9.63%) required ICU support during their hospital stay. At least two or more observers reported a typical and indeterminate COVID-19 pneumonia CT pattern according to RSNA categorization in 70% (285/405) of patients. Among these, 63% (179/285) had a positive polymerase chain reaction (PCR test for the SARS-CoV-2 virus. The median number of lobes involved according to CT was higher in patients who required ICU support (median interquartile range [IQR], 5[3; 5] vs. 3[0; 5]). The median ZCVS score was higher among the patients that subsequently required ICU support (median [IQR], 4[0; 12] vs. 13[5.75; 24]). The bootstrap comparisons of AUC ROC showed significant differences between reporting tools, and the ZCVS was found to be superior (AUC ROC, 71-75%). The ZCVS score at the first admission showed a linear and significant association with adverse outcomes among patients with the lower respiratory tract symptoms during the COVID-19 pandemic.

## INTRODUCTION

In December 2019, a cluster of pneumonia cases emerged in Wuhan, China [1]. Molecular studies identified a novel coronavirus distinctly related to the severe acute respiratory syndrome coronavirus (SARS-CoV-2) [2]. The disease caused by the virus, eventually named coronavirus disease 2019 (COVID-19), rapidly spread over continents leading to a pandemic. As of November 2, 2020, 47 million laboratory-confirmed cases had been reported globally, including more than 1 million fatalities.

Viral nucleic acid testing is playing an indispensable role in the diagnosis and prevention of the spread of the SARS-CoV-2 [3]. However, nucleic acid testing has rigorous laboratory specifications and qualitative result at least is not related to the severity of the disease. Moreover, the sensitivity of available molecular tests varies according to the type and quality of the sample [4]. Computed tomography (CT) imaging has been successfully used in the detection of lung lesions classification of severity among COVID-19 patients [5].

Early institution of anti-viral drugs and appropriate supportive measures is central in the management of COVID-19 infection. The sensitivity of chest CT imaging among COVID-19 patients is exceptionally high [6]. Therefore, it has been suggested that the CT imaging can be used to aid the diagnosis of COVID-19 infection until the results of molecular tests are available [7].

During the pandemic, hospitals have reorganized to manage the excessive numbers of patients [8]. Effective triaging according to disease severity and identification of patients with potential adverse outcomes is vital. We believe that CT imaging can substantially contribute to predicting adverse outcomes at admission and therefore has a valuable role in the allocation of such patients to appropriate units. However, data regarding the predictive performance of CT imaging is nil or sparse.

In this study, we evaluate the performance of CT reporting tools in predicting adverse outcomes among patients hospitalized due to the lower respiratory tract infections during the COVID-19 pandemic.

## MATERIALS AND METHODS

### Study population and definitions

This retrospective study was carried out in two different tertiary care centers. Ethics committee permission was obtained from the university local ethics committee (Decision number: E1-20-438). Medical records of the two hospitals for the timeline from March 15 to April 20, 2020, were reviewed retrospectively. A total of 405 patients suspected to have COVID-19, admitted with severe respiratory tract symptoms (any of respiratory rate >23, O2 Saturation <94%, and shortness of breath) and subsequently underwent chest CT and real-time polymerase chain reaction (RT-PCR) according to the national standard protocol published by the Ministry of Health were included in the study. Patients with incomplete clinical or laboratory information, history of trauma, and post-contrast CT images were excluded from the study.

Intensive care unit (ICU) admission was the primary outcome parameter. Patients requiring high-level respiratory support, mostly through invasive means, are admitted to the ICU. Following ICU admission, variables determining the outcome of the patient escalate with the contribution of undetermined confounders. Therefore, ICU admission was deemed as a more precise outcome measure.

### CT protocol

CT examinations were performed either with a 16-slice GE Optima CT520 (300 patients) or a 128-slice GE Revolution EVO (105 patients).

The scanning parameters were as follows: Tube voltage, 100 kV if bodyweight ≤80 kg and 120 kV for patients >80 kg; tube current, 70-120 mAs with automatic dose modulation, and slice thickness of 1.25 mm. The radiation dose of chest CT was as follows: Volume CT dose index, 3.45-5.60 mGy; effective dose, 1.4-2.7 mSv if bodyweight ≤80 kg or >80 kg, respectively.

Images were obtained with mediastinal (width, 400 HU; level, 40 HU) and parenchymal (width, 1600 HU; level, −450 HU) window settings. After imaging in the axial plane, coronal and sagittal plans were created by performing multiplanar reconstruction.

### CT images analysis

The images were reviewed independently by three experienced radiologists with 5 years, 10 years, and 11 years of experience in chest imaging and two resident radiologists. The reviewer radiologists were blinded to the patients’ personal information, RT-PCR results, epidemiological history, and clinical characteristics.

The Zonal CT Visual Score (ZCVS) has previously been applied during SARS and MERS epidemics [9,10]. CT imaging was used to evaluate the extent of lung involvement. Each lung was primarily divided into the upper zone (from the apex to above the carina level), middle zone (from the carina to the inferior pulmonary vein), and the lower zone (below the inferior pulmonary vein). Each lung was further divided into two parts: Anterior and posterior, by a vertical line crossing the midpoint of the diaphragm in a sagittal position. Therefore, each lung was divided into six zones; the upper anterior zone, the upper posterior zone, the middle anterior zone, the middle posterior zone, the lower anterior zone, and the lower posterior zone. In each lung zone, the percentage of the involved area was scored as score 0 (no involvement), score 1 (<25% involvement), score 2 (25% to <50% involvement, score 3 (50% to <75% involvement), or score 4 (>75% involvement).

The scores were recorded for different lung regions, and a sum of all the lung regions was calculated. The score ranges between a maximum of 48 and a minimum of zero.

The number of involved lobes was also noted. Each lobe was evaluated separately. The final score was the number of involved lobes (0-5) [11].

**FIGURE 1:**
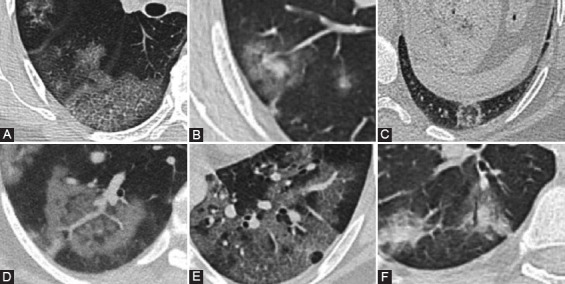
Computed tomography features of coronavirus disease 2019 pneumonia. (A) Reticular pattern. (B) Halo sign (Ground-glass opacities with centrally consolidation). (C) Reverse halo sign (a ground-glass opacity area in the center and completely surrounded by high-density consolidation). (D) Vascular enlargement. (E) Air-bubble sign. (F) Air bronchogram appear on the background of consolidation and ground-glass opacities.

The main lung manifestations on CT that were recorded are as follows: Ground glass opacity (GGO), consolidation, reticular pattern (thickened interlobular and intralobular septum based on GGO, showing crazy-paving sign); air bronchogram, halo sign (the ground-glass shadow around the nodule), reverse halo sign (GGO in the center, surrounded by high-density consolidation shadow), vascular dilation, and air-bubble sign [12-14]. In addition, mediastinal lymph nodes, thickening of the pleura, pleural effusion were included and noted as extrapulmonary findings (Figure 1) [15].

If the axial distribution was prevalent in the outer third of the lung, it was classified as peripheral, and if it was predominant in the inner two-third, it was classified as central. It was classified as diffuse if both central and peripheral distribution were present.

Abnormality patterns on CT for diagnosis of COVID-19 were categorized according to the classification of Radiological Society of North America expert consensus statement as (1) typical (Even if it is not certain for COVID-19 pneumonia; high specificity; and common imaging features); (2) indeterminate (Findings reported in COVID-19 pneumonia, but not specific); (3) atypical (Features that suggest alternative diagnoses rather than COVID-19 pneumonia); and (4) negative for pneumonia [16].

### Statistical analysis

Statistical analysis was accomplished on open source platform R (R Foundation for Statistical Computing, Vienna, Austria). The analysis was conducted with appropriate packages such as “rms”, “pROC,” and “compare Groups.”

Continuous data, if normally distributed, were presented as means; otherwise, they were presented as medians and quantiles. Normality was assessed with the Shapiro–Wilk test. In univariate comparisons, the Student’s t-test or Wilcoxon rank-sum test was applied according to the normality assumption of continuous data. Dichotomous data were compared using the Chi-square test or Fishers’ exact test where appropriate. Interobserver agreement was analyzed using Light’s Kappa (k) statistics.

Multivariate logistic models were constructed including medically important variables plus variables found significant in univariate comparisons (*p* < 0.05). Missing observations were multiply imputed, and individual estimates were pooled. Models were internally validated using the bootstrap technique. Model performances were compared with C-index and Somer’s Dxy rank discrimination index. A nomogram was constructed using the “rms” package.

The performance of CT reporting tools was tested using receiver operating characteristic (ROC) curves and compared area under the curve (AUC) of tools with statistical tests based on bootstrapping. We fitted a Bayesian random intercept model using “brms” package which fits models with Stan related sampling algorithms. Final model included the CT score values, O2 saturation at admission, age, and PCR test positivity. Results were expressed as mean 80% high density intervals.

## RESULTS

### Clinical characteristics

A total of 405 patients hospitalized due to the lower respiratory infections during the COVID-19 pandemic were enrolled in this study of whom 184 (45.4%) were female, and 221 (54.6%) were male, with the age range of 35-65 years and a mean age of 52 years (Table 1). The median number of days from onset of symptoms to CT examination was 5 days [interquartile range (IQR), 3-7]. The patients had various clinical symptoms and laboratory findings. In 203 patients, at least one nucleic acid test for COVID-19 was positive and no statistical difference was found in the incidence of comorbidity between COVID-19 and non-COVID 19 patients (Table 2). The lymphocyte count was reduced in 85/305 (28%) patients. While the median oxygen saturation was 95% at the time of admission, the median respiratory rate was 20 per min.

**TABLE 1:**
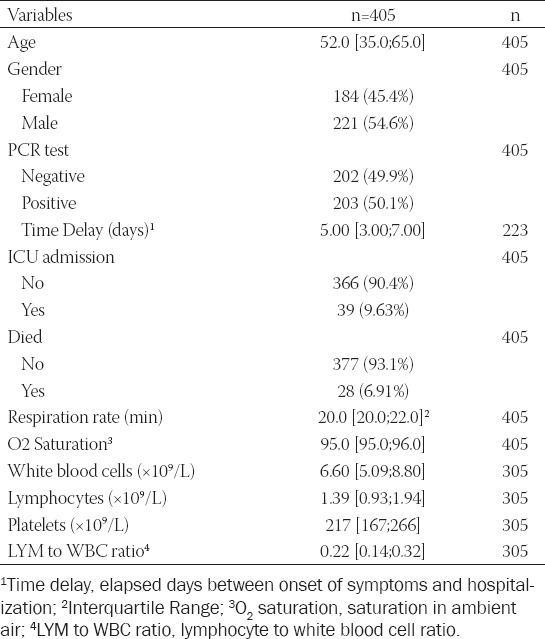
Baseline descriptive characteristics of the cohort

**TABLE 2:**
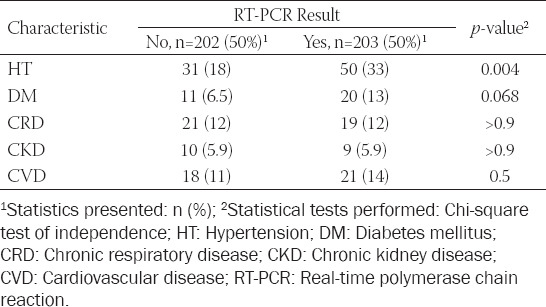
Frequencies of comorbidities according to RT-PCR Results

The ICU group included 39/405 (9.63%) patients. Patients who admitted to the ICU were older (median, 65 [52; 72] vs. 50 [34; 64], *p* < 0.001) and had a higher hypertension proportion in comparison to those without ICU admission (Table 3). Twenty-eight of 405 patients deceased.

**TABLE 3:**
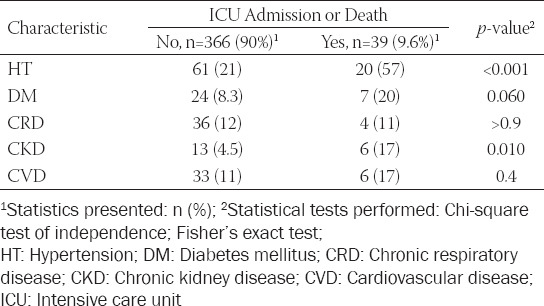
The relationship between the frequencies of comorbidities and ICU admission

### CT findings

At least two or more observers reported a typical and indeterminate COVID-19 pneumonia CT pattern in 285/405 (70%) of the patients. Among these, 63% (179/285) had a positive PCR test for the SARS-CoV-2 virus. The main CT density changes were reported in varying percentages among observers. Accordingly, ground-glass opacities (GGO) were detected in a minimum of 66% to a maximum of 73%; whereas consolidation was seen in a minimum of 34% to a maximum of 44%, respectively. Other lung manifestations were also seen at different rates: Reticular pattern 30%-38%; air bronchogram 23%-26%, halo sign 5%-30%, reverse halo sign 5%-14%, vascular dilation 13%-40%, and air-bubble sign 3%-5%.

In terms of CT characteristics, mediastinal and hilar lymph nodes were observed in 2-30%, pleural effusion was observed in 6-8% of patients, and pleural thickening was observed in 5-8% of patients, varying between observers.

The incidence of consolidation (79.5% vs. 40.7%), air bronchogram (53% vs. 23%), reticular pattern (48.7% vs. 27.6%), and GGO (87.2% vs. 70.8%) in patients who needed ICU care was higher than that of the patients who did not need ICU. Besides, patients who needed ICU showed a higher incidence of pleural effusion than patients who did not need ICU (31% vs. 3%).

The median number of lobes involved on CT was higher in patients who subsequently required ICU (median [IQR], 5[3; 5] vs. 3 [0; 5]). The initial chest CT studies of the 39 patients with ICU showed that disease affected all five lobes in 22 (56%) patients.

Lower zone and posterior predominance (284/405, 70% and 290/405, 72%, respectively) and diffuse distribution on the transverse plane (227/405, 56%) were the most common distribution patterns. Both lower lobes were involved in 228/405 patients.

**FIGURE 2:**
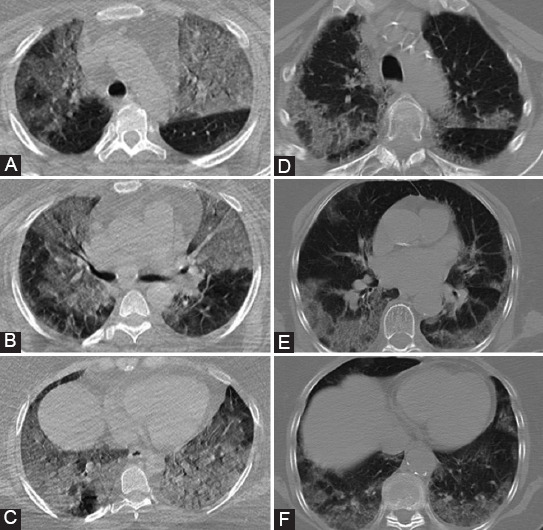
(A-C) 40-year-old woman with coronavirus disease 2019 (COVID-19) pneumonia. Non-contrast computed tomography (CT) was performed on day of admission. Chest CT images showed large areas of bilateral consolidation and ground-glass opacities in bilateral lungs and air bronchogram. The lesions involved in five lung lobes and the Zonal CT Visual Score was 44. Patient was admitted to the intensive care unit (ICU). (D-F) Axial CT images of a 72-year-old woman with COVID-19 pneumonia revealed bilateral consolidation, ground-glass opacities with mainly peripheral distribution, and accompanying reversed halo sign. The lesions involved in five lung lobes and the Zonal CT Visual Score was 27. Patient was admitted to the ICU.

**FIGURE 3:**
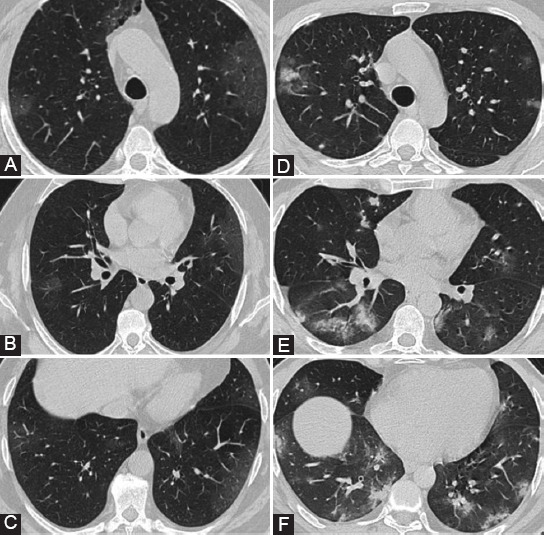
(A-C) Axial computed tomography (CT) images of a 39-year-old man with coronavirus disease 2019 (COVID-19) pneumonia showed bilateral ground-glass opacities with dominantly peripheral distribution. All lung lobes were involved and the Zonal CT Visual Score was 14. Patient was discharged from the hospital without intensive care unit (ICU) admission. (D-F) 67-year-old man with laboratory confirmed COVID-19 pneumonia. Axial chest CT images demonstrated bilateral consolidation, ground-glass opacities, halo sign, and vascular enlargement. The lesions involved in five lung lobes and the Zonal CT Visual Score was 16. He recovered without ICU admission.

No significant difference was observed between the median CT scores for the left lung and right lung; the scores for both were 2 [0; 6] to 3 [0; 6]. The median CT score was higher in patients who required ICU (median [IQR], 4 [0; 12] vs. 13 [5.75; 24]) (Figures 2 and 3).

### Predictive performances of reporting tools

**FIGURE 4:**
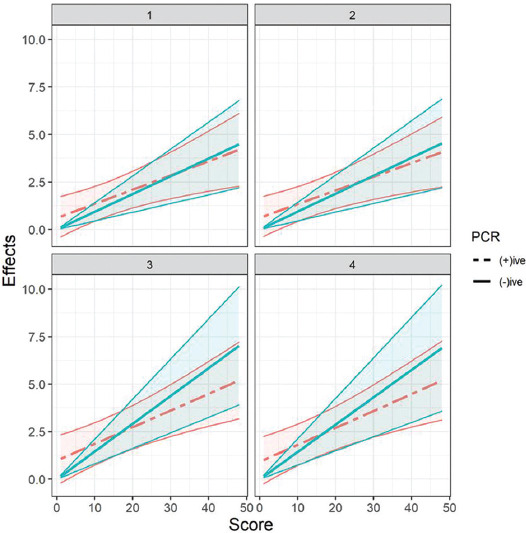
Estimated effects of Zonal computed tomography visual score among coronavirus disease 2019 polymerase chain reaction positive and negative patients for observers.

We obtained effect estimates from logistic models. Interobserver agreement Light’s k value was found in a substantial agreement range (k = 0.706). The effect estimates between observers were in considerable agreement (Figure 4). More importantly, the direction of estimates for ZCVS was not different between PCR positive and negative patients. Table 4 displays predictive performances of three tools based on the area under the ROC (AUC ROC) statistics. The bootstrap comparisons of AUC ROC showed significant differences between reporting tools, and the ZCVS was found to be the best predictive tool. The final random intercepts logistic model revealed posterior high-density intervals significant for other variables, which suggests that data from future studies would also be favorable for these variables in terms of predicting adverse outcomes.

**TABLE 4:**
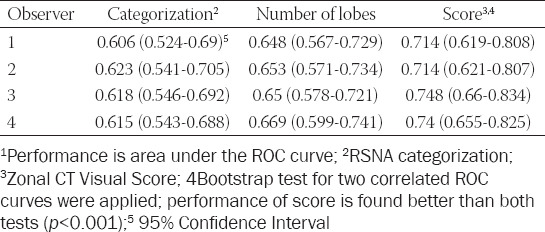
Discrimination performances of methods for outcome (need for ICU support)^1^

**FIGURE 5:**
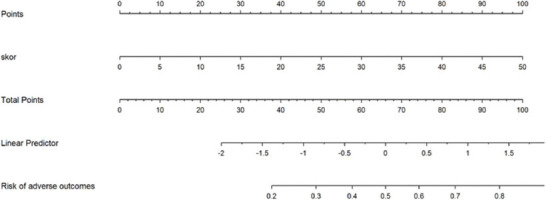
A nomogram to calculate the predicted risk of adverse outcomes manually according to Zonal Computed Tomography Visual Score.

Figure 5 displays a nomogram constructed by estimates from a logistic model. The linear relationship between the score and outcome, here the need for ICU support, is visually seen. The nomogram aids in calculating the predictive probabilities. Patients with a ZCVS of 20 had a 20% possibility of going to the intensive care unit. In contrast, in patients with a score of 45, this rate was above 80%, regardless of clinical and demographic parameters.

## DISCUSSION

The results of the present study show that the ZCVS obtained in the emergency department successfully predicts adverse outcomes for these patients. According to our findings, the ZCVS value shows high inter-observer agreement and success in outcome prediction. As the BT score increases, the probability that the patient will need ICU increases. This knowledge will help guide the management of patients and determine the appropriate treatment.

Several CT scores based on parenchymal involvement have been previously reported for the assessment of disease severity in COVID-19 and other respiratory system infections [17]. However, limited data are available regarding the prognostic value of the first CT in predicting adverse outcomes such as intensive care hospitalization [18]. Zhou et al. calculated the involvement of the lung by scoring 0 to 5 points for each lobe on CT images [19]. They demonstrated that the CT scores of the patients in progressive-stage were significantly higher than those of the patients in early stage in 62 confirmed COVID-19 patients. We calculated the zonal score differently to evaluate not only the craniocaudal distribution but also the anteroposterior distribution. We applied this score to patients with severe respiratory symptoms to correlate with adverse outcomes.

The previous studies have shown that COVID-19 pneumonia requires ICU admission in up to 17% of patients and the mortality ranges from 11% to 15% [1,20,21,22]. Considering that ARDS is the primary cause of poor prognosis and death in patients with COVID-19 pneumonia and many other respiratory pathologies, we hypothesized that the volume of involvement of the lung in the first CT might predict the outcome of the disease. We tested the patients at the time of presentation to the emergency room to predict the probability of subsequent admission to the ICU. If the CT score exceeded 32 points, the probability of ICU admission was over 50%, which has not been previously reported in the literature.

In patients who died of COVID-19, the presence of protein and fibrous exudates and bilateral diffuse alveolar damage was higher in histological examinations of lung biopsy specimens. These pathological changes have various manifestations on chest CT including GGO and consolidation [23,24]. Therefore, as found in the present study, patients with adverse outcomes develop a high proportion of ground glass and consolidation in a large number of lobes. It was also found in this study that the number of affected lobes provided information in terms of outcome but was not as successful as the Zonal CT Visual Score. The estimation of the outcome by the number of involved lobes was 65% successful, while the CT score was 74% successful. Colombi et al. reported that more than 3 lobe involvement was worse in terms of adverse outcomes [25].

The previous studies have shown that different viral infections produce pathological and CT results similar to COVID-19 [26]. The damaged pulmonary epithelial cells, hyaline membranes, and a large number of tissue cells and mass thrombi formed by proliferative fibrous tissue that blocks the small airway and air cavity seems to be the primary pathological basis of the CT findings, especially the GGO [27]. The results of GGO, consolidation, reticular pattern, air bronchogram, and vascular enlargement that we evaluated in our study are not specific for COVID-19, and non-COVID 19 illnesses lead to similar CT findings and can cause a discrepancy between categories.

The most important limitations in this study are the low sensitivity of PCR in these patients at the time of testing and that the PCR tests of other respiratory viruses could not be studied due to the laboratory workload. Future studies with a comprehensive analysis of all possible viral pneumonia etiologies would validate the performance of ZCVS.

## CONCLUSION

The rate of Zonal CT Visual Score at the first admission may predict the subsequent requirement of ICU admission and facilitate the clinician decision for respiratory support in the rush of the pandemic in patients with the lower respiratory tract symptoms. Quantitative evaluation of the extent of lung involvement may be useful for routine patient management in emergency departments.
